# Prevention of AcuTe admIssioN algorithm (PATINA): study protocol of a stepped wedge randomized controlled trial

**DOI:** 10.1186/s12877-021-02092-2

**Published:** 2021-02-27

**Authors:** Anders Fournaise, Jørgen T. Lauridsen, Mickael Bech, Uffe K. Wiil, Jesper B. Rasmussen, Kristian Kidholm, Kurt Espersen, Karen Andersen-Ranberg

**Affiliations:** 1 Department of Cross-sectoral Collaboration, Region of Southern Denmark, Damhaven 12, 7100 Vejle, Denmark; 2 Epidemiology, Biostatistics and Biodemography, Department of Public Health, University of Southern Denmark, 5000 Odense, Denmark; 3 Geriatric Research Unit, Department of Geriatric Medicine, Odense University Hospital, J. B. Winsløws Vej 4, 5000 Odense, Denmark; 4 Department of Business and Economics, University of Southern Denmark, Campusvej 55, 5000 Odense, Denmark; 5 The Danish Center for Social Science Research (VIVE), Herluf Trolles Gade 11, 1052 Copenhagen, Denmark; 6 The Maersk Mc-Kinney Moller Institute, University of Southern Denmark, Campusvej 55, 5000 Odense, Denmark; 7 Centre for Innovative Medical Technology, Odense University Hospital, J. B. Winsløws Vej 4, 5000 Odense, Denmark; 8 Danish Ageing Research Center, University of Southern Denmark, J. B. Winsløws Vej 9B, 5000 Odense, Denmark

**Keywords:** Geriatrics, Community-dwelling, Older people, Acute admission, Algorithm, Decision support, Stepped-wedge cluster randomized controlled trial, Home care

## Abstract

**Background:**

The challenges imposed by ageing populations will confront health care systems in the years to come. Hospital owners are concerned about the increasing number of acute admissions of older citizens and preventive measures such as integrated care models have been introduced in primary care. Yet, acute admission can be appropriate and lifesaving, but may also in itself lead to adverse health outcome, such as patient anxiety, functional loss and hospital-acquired infections. Timely identification of older citizens at increased risk of acute admission is therefore needed.

We present the protocol for the PATINA study, which aims at assessing the effect of the ‘PATINA algorithm and decision support tool’, designed to alert community nurses of older citizens showing subtle signs of declining health and at increased risk of acute admission. This paper describes the methods, design and intervention of the study.

**Methods:**

We use a stepped-wedge cluster randomized controlled trial (SW-RCT). The PATINA algorithm and decision support tool will be implemented in 20 individual area home care teams across three Danish municipalities (Kerteminde, Odense and Svendborg). The study population includes all home care receiving community-dwelling citizens aged 65 years and above (around 6500 citizens). An algorithm based on home care use triggers an alert based on relative increase in home care use. Community nurses will use the decision support tool to systematically assess health related changes for citizens with increased risk of acute hospital admission.

The primary outcome is acute admission. Secondary outcomes are readmissions, preventable admissions, death, and costs of health care utilization. Barriers and facilitators for community nurse’s acceptance and use of the algorithm will be explored too.

**Discussion:**

This ‘PATINA algorithm and decision support tool’ is expected to positively influence the care for older community-dwelling citizens, by improving nurses’ awareness of citizens at increased risk, and by supporting their clinical decision-making. This may increase preventive measures in primary care and reduce use of secondary health care. Further, the study will increase our knowledge of barriers and facilitators to implementing algorithms and decision support in a community care setup.

**Trial registration:**

ClinicalTrials.gov, identifier: NCT04398797. Registered 13 May 2020.

## Background

The challenges imposed by demographic changes will confront health care systems in the years to come [1]. It is a global concern that even highly effective health care systems will struggle with meeting the demands of ageing populations [2], as higher age is associated with multimorbidity, health deterioration with functional decline, and subsequent increased utilization of health care services [3–6].

In older citizens acute hospitalization can be highly necessary and lifesaving, but may also lead to adverse consequences, such as hospital-acquired infections, anxiety and distress, poorer functional health, and death [3, 7–10]. Prevention of acute admission is therefore exceptionally important in higher age groups, but requires timely detection of disease symptoms, functional and mental deterioration and health care interventions. However, early recognition of disease is hampered by diagnostic challenges following older citizens’ higher prevalence of multimorbidity, polypharmacy, functional impairment, and social issues, which altogether yield complex interactions and increases the risk of mismanagement [11]. In addition, atypical presentation of symptoms may delay timely diagnosis, which is why novel predictive tools are needed for timely recognition of older citizens at increased risk of acute disease and subsequent acute hospitalization [12].

Prediction models using data from electronic health records to identify those in the highest risk of acute hospitalization have been increasingly studied. A systematic review from 2014 identified 27 prediction models for acute admissions. However, only half of the models were targeted older citizens, and in addition, the predictive models in general required large administrative or clinical data sets, which were analyzed retrospectively [13]. Most models are based on data from electronic hospital records and many have been found effective in predicting risk of acute admissions [13–19]. Only a few studies have focused on prediction models solely based on home care data [20], and very few studies have implemented and tested a prediction model in practice, mainly due to ethical and economic considerations [21], barriers among professionals, such as trust in the technology [22], and the fact that data on health and care is registered primarily for the use and support for health professionals, not for input to algorithms [23]. Thus, datasets are more often designed for retrospective analysis than for prospective use [24].

In a tax-funded (Beveridgian) health care system free of charge for health care receivers utilization of health and home care services mirrors older citizen’s overall health [25]. An increased need of health care may be the first sign of emerging acute disease. In an earlier retrospective study, we found a significant increase in municipal home care service (hours/week) over a 12-month period prior to acute hospitalization [3]. Based on this finding we have developed the Prevention of AcuTe admIssioN Algorithm (PATINA), a novel predictive model that analyzes administrative data on home care utilization and yields a warning to community nurses (hereafter referred to as nurses) about citizens at increased risk of acute hospitalization. Further, to assist nurses in their assessment of citizens identified by the algorithm as being ‘at risk’, a decision support tool has been developed. The intervention is presented in the method section.

The primary objective of the PATINA project is to implement and evaluate the combined effect of the ‘PATINA algorithm and the decision support tool’ on the prevention of acute hospital admissions of older community-dwelling citizens [3]. Further, we will evaluate the effects on citizens utilization of health care service in the primary and secondary health care sector and estimate costs. A second objective is to investigate nurses’ role in achieving the desired effects of the implementation, by linking employees’ motivation and attitudes toward the ‘PATINA algorithm and the decision support tool’ to the primary and secondary outcome of the study.

In this paper, we present the study protocol for the PATINA project, where we use a stepped-wedge randomized controlled trial, explaining the methods, design and intervention of the study. The study protocol (version 1.2, 13 May 2020) follows the SPIRIT statement [26] and has been adapted to suit the format of an article.

## Methods/design

### Study design

**Fig. 1:**
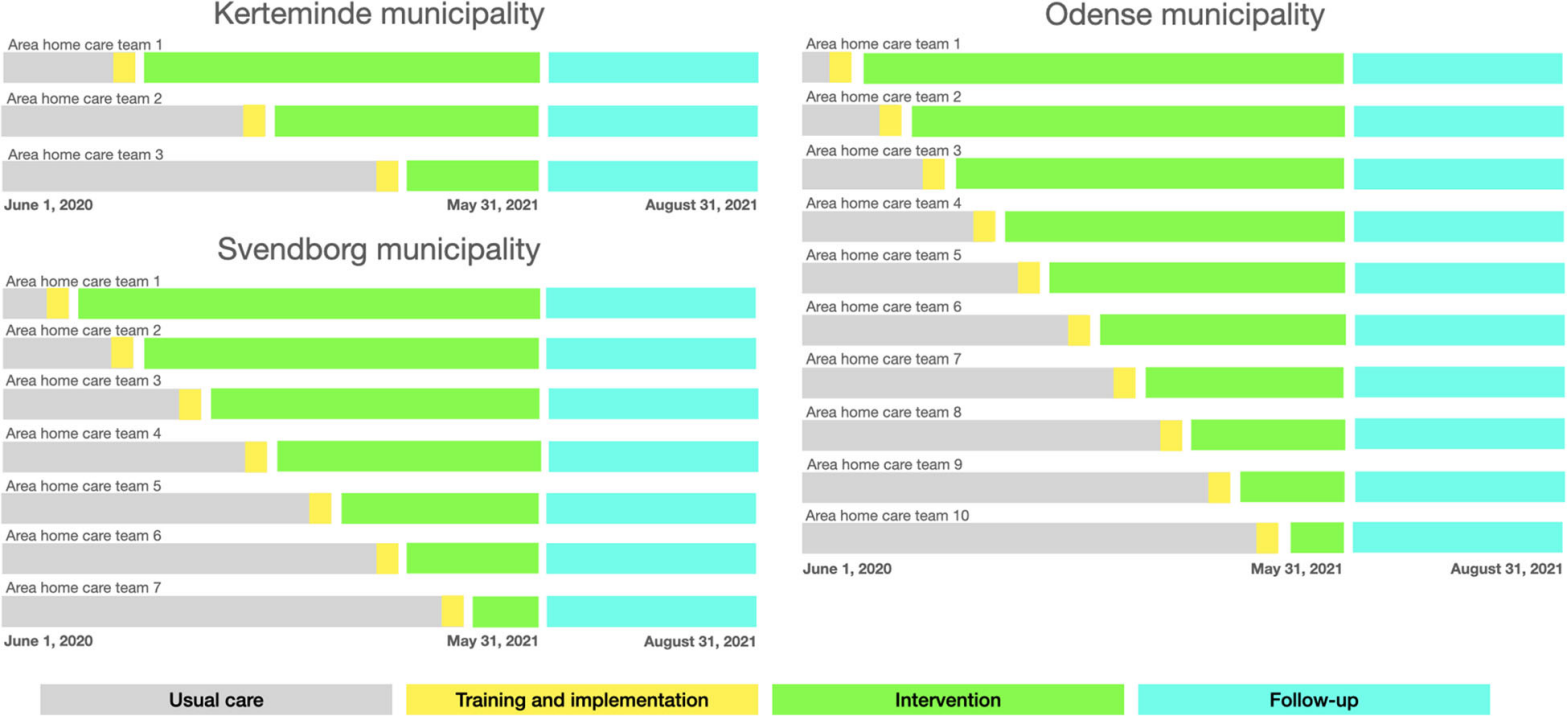
The PATINA study stepped-wedge design in three municipalities with three (Kerteminde), seven (Svendborg) and 10 (Odense) area home care teams. With a scheduled randomized inclusion of an area home care team every twelfth week in Kerteminde, every sixth week in Svendborg and every fourth week in Odense Municipality. With a one- and three-month follow-up

The study is designed as a multicenter randomized controlled trial using a stepped-wedge cluster design (Fig. 1) to implement and test the ‘PATINA algorithm and decision support tool’ in three Danish municipalities. Three stages will sequentially be rolled-out across the area home care teams in the three municipalities (described later); usual care exposure; intervention implementation and intervention exposure, followed by a one month and three-month post intervention follow-up. The timing of the one-way crossover from usual care to intervention exposure is randomly allocated during the intervention period (June 1, 2020 – May 31, 2021).

#### Setting and study population

Denmark has 5,8 million inhabitants (2020), of which 264.000 are 80 years or older, a number forecasted to increase to 670.000 in 2057 [27]. Like most Nordic countries the Danish public welfare system is tax funded and builds on a societal consensus that the public sector is responsible for providing good and equal health and social care [28, 29]. Health care, i.e. hospital treatment, consultations with a primary care physician or specialist physician, and home care, is universal and free of charge at the point of utilization [30]. Health care services are provided by five regions, who are responsible for operating and funding hospitals and financing primary care physicians and specialist physicians, while the 98 municipalities are responsible for providing home care and social care, care in care homes, nursing care, training and rehabilitation, health promotion and more [30]. Municipalities strive to care for older citizens in their own home as long as possible. Municipal health care services are allocated based on the citizen’s functional level and ability of self-care and may be adjusted following changes in an individual’s self-care ability. Home care, nursing care and rehabilitation services are all delivered by the municipal area home care teams, which are organized in teams covering specified geographical areas. Unlike many other countries nursing care in the community is thus delivered by the municipalities and not by a national health service [30].

The trial is carried out in three Danish municipalities of different sizes, populations, and organizational characteristics: Kerteminde as the smaller, Svendborg as the medium sized and Odense as the larger municipality (see description in Table 1). In each municipality we include area home care teams (20 in total for all three municipalities), that primarily have a focus on delivering health care services to community dwelling older citizens. Two additional area home care teams in Odense Municipality have already been excluded as their primary focus is younger citizens with mental disorders, not older citizens.

**Table 1:** Characteristics of the three intervention municipalities in the PATINA study [27]. OADR: Old Age Dependency Ration (OADR), ratio between the number of citizens aged 65+ years and the number of persons in the working age 15–64

	Kerteminde	Svendborg	Odense
Size, km^2^	207	417	305
Population, N	23,833	58,355	205,881
Population above 65 years of age, n	6142	14,206	35,811
Population above 80 years of age, n	1590	3407	9012
Old age dependency ration (OADR), 15–64/65+ years	0.43	0.39	0.25
Organizational characteristics of area home care teams	Geographical unites	Geographical unites	Unites organized by mental and somatic disease characteristics and geography
Number of area home care teams (included in the study)	3 (3)	7 (7)	12 (10)

The study population will include all community-dwelling citizens above 65 years of age, who receive home care in Odense, Kerteminde and Svendborg Municipalities.

#### Intervention

The intervention is the ‘PATINA algorithm and the decision support tool’, which systematically guides nurses in their overall health assessment of citizens flagged by the algorithm.

#### PATINA algorithm

The algorithm monitors and analyzes community-dwelling older citizens’ use of municipal home and nursing care with almost real time data (one-week delay). It uses individual administrative data on assigned amount of time (minutes per week) for home care, practical (domestic) help, training/rehabilitation, and nursing care delivered to each citizen.

**Fig. 2:**
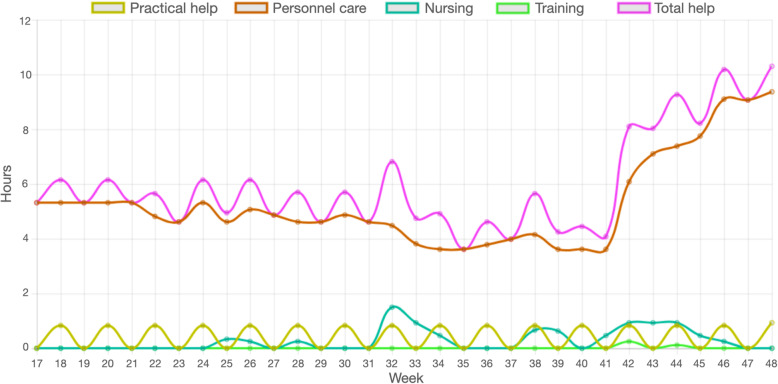
Graph from the PATINA algorithm visualizing a citizen’s utilization of home care (practical help and personnel care), nursing, training and total help

In each of the three municipalities, a weekly data set with information on health care use during the past week is fed into three separate secure databases via a front-end upload module. Data is then analyzed by the algorithm by comparing home care utilization the last month to a prior period of six months. The algorithm then produces a list of citizens that have triggered the threshold configured for the algorithm. A graph visualizing each citizens utilization of personal care, practical help, training/rehabilitation and nursing care during the last six months is also produced, see Fig. 2.

In the intervention phase, nurses in the included area home care teams receive the list of citizens that have surpassed the threshold. These are considered to be at increased risk of adverse health outcomes and subsequently of acute hospitalization. The list also provides graphs visualizing each citizens’ health service utilization. Simultaneously, the algorithm sends the information to a research database setup in Redcap [31, 32], which then forwards a unique link for each citizen to an allocated community nurse. The link gives the nurse access to the ‘PATINA decision support tool’. The tool is designed as a questionnaire, which is covered and filled-in by the nurse.

#### PATINA decision support tool

The PATINA decision support tool is designed to nudge nurses to reflect upon more recent notifications in the electronic care records regarding changes in health and need of care. Such changes may be subtle signs of health deterioration, and not just ageing processes. Additionally, the ‘PATINA decision tool’ includes validated health assessment scales and information on risk factors of acute disease, such as functional level (Barthel-20) [33], frailty (Clinical Frailty Scale) [34, 35], Brief Geriatric Assessment [36], medications, falls tendency, weight, mental state, dehydration, and more. Further, the tool supports and covers areas of health care, which nurses in Denmark are obligated to assess regularly, e.g. assessment of citizens general state of health [37].

Nurses complete the decision support workflow by reviewing information in the citizen’s electronic care record and apply their knowledge about the citizen from their daily work. After assessing a ‘citizen at risk’ using the decision support tool, the data is sent to the research database and a PDF file containing the assessment is automatically forwarded to the nurse via email. The PDF file is then saved on the citizen’s electronic health record for later use by the nurse and other colleagues, as well as for reference.

Data from the citizen can trigger the algorithm’s alarm for several weeks in a row. The first time a citizen appears on the list the nurse must assess the citizen’s current health and functional situation using the PATINA decision support tool. When a citizen continues to be on the list, the nurse compares the citizens actual health care situation with the latest ‘PATINA decision tool assessment’, thereby judging whether the citizen’s situation has worsened, and further action is needed. In this case the citizen is assessed once more using the decision tool and a PDF file is saved as a new reference point.

**Fig. 3:**
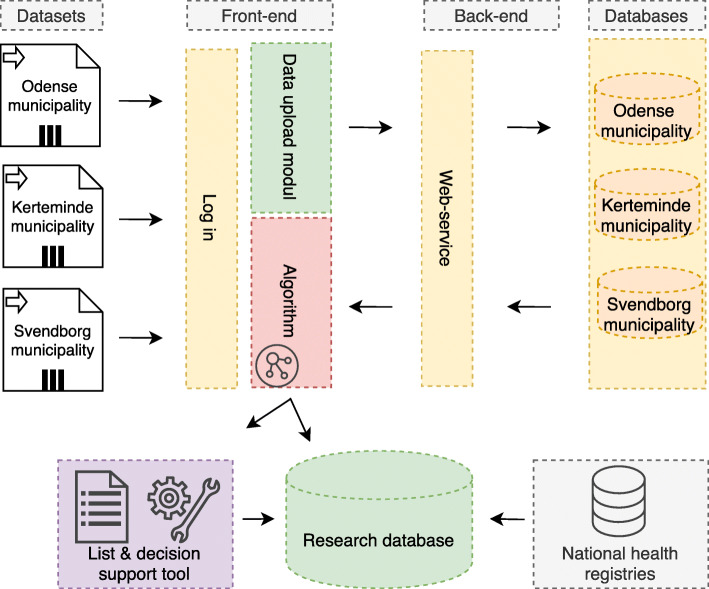
The IT-ecosystem and dataflow in the PATINA algorithm and decision support tool. The figure was created using draw.io (open-source freeware)

The IT ecosystem and data flow of the project are visualized in Fig. 3. A more detailed description of how the algorithm has been developed, IT infrastructure and security, and how the threshold was configured, will be presented later in a separate paper.

### Study procedures

#### Randomization and implementation

The stepped-wedge design allows all 20 area home care teams across the three municipalities to receive the intervention, but at different time points according to randomization. Following the time schedule, a new area home care team is included in Kerteminde Municipality approximately every 12 weeks, in Svendborg Municipality every six weeks, and in Odense Municipality every four weeks (Fig. 1). The computer-generated stepwise randomization is carried out by a statistician from Open Patient data Explorative Network (OPEN), Odense University Hospital, Region of Southern Denmark.

All three municipalities are made aware of the randomization procedure prior to study start. However, the starting date for the intervention exposure will not be announced until four weeks prior to implementation start.

In this study it is not possible to blind neither nurses or other health care professionals, nor the management in each municipality. However, nurses and the management are instructed only to talk about the project with coworkers within their own area home care team, and not with colleagues in other teams.

#### Study outcome

The primary outcome of the study is acute hospital admission. Secondary outcomes cover readmission, preventable admission, contacts to outpatient clinics, death as well as changes and costs in health care utilization. Further, we will investigate barriers and facilitators for community nurse’s acceptance and use of the algorithm using data gathered in a three-wave survey sent to nurses in the individual area home care teams, which will be integrated in the SW-RCT design. The study outcomes and outcome measures are presented in Table 2.

**Table 2:** Project outcomes and outcome measures in the PATINA study

	Outcome	Outcome measure
***Primary outcome***		
Outcome 1	Acute admission	Proportion of acute hospital admissions after being identified at risk by the algorithm
***Secondary outcomes***		
Outcome 2	Preventable admission	Preventable admission after being identified at risk by the algorithm
Outcome 3	Readmission (30-days)	Readmission to a Danish hospital within 30 days from a hospital discharge
Outcome 4	Outpatient contacts	Proportion of contacts to outpatient clinics after being identified at risk by the algorithm
Outcome 5	Primary care physicians contacted	Contacts to a primary care physician after being identified at risk by the algorithm
Outcome 6	Death (90-days)	Proportion of diseased citizens identified at risk by the algorithm within 90 days
***Health care utilization and economic outcomes***		
Outcome 7	Changes in utilization and costs of municipal home, nursing and social care services	Information on municipal home, nursing and social care services will be collected as part of the data used in the PATINA algorithm. Costs will be summed from the first date a citizen is identified at risk by the algorithm
Outcome 8	Changes in utilization and costs of planned and unplanned hospital treatment	Information on hospital treatment (planned and unplanned) will be collected from the Danish National Patient Registry. Costs will be summed from the first date a citizen is identified at risk by the algorithm
Outcome 9	Changes in utilization and costs of contacts to primary care physicians	Information on contacts to primary care physicians will be collected from the Danish National Health Insurance Service Register. Costs will be summed from the first date a citizen is identified at risk by the algorithm
Outcome 10	Cost of implementation of the PATINA algorithm and decision support tool	The cost of implementing the algorithm and decision support tool will be calculated as time total time spent for introduction and working with the algorithm for each nurse
***Process outcomes***		
Outcome 11	Barriers and facilitators for nurse’s acceptance and use of the algorithm	Data will be collected as a three-wave survey, which will be integrated in SW-CRT design and follow the step-wise implementation of the algorithm in each of the tree municipalities

#### Recruitment and enrollment

The management in each of the three municipalities have approved their participation in the study. To support the project manager, a project coordinator has been appointed in each municipality. All community nurses and their immediate superior have participated in an information meeting, with the purpose of explaining the background of the study, enrollment and training procedures. Information about the PATINA algorithm and decision support tool is kept to a minimum.

When an area home care team is randomized to receive the intervention the project manager (AF) and project coordinator meet with the community nurses that are selected for participation in the intervention. The meeting has a scheduled time frame of 2 h and is held twice, seven days apart. The purpose of the first meeting is to introduce the nurses to the background of the study, their role and introduction to the PATINA algorithm and decision support tool. In the second meeting, nurses will complete a practical training session using the PATINA algorithm and decision support tool.

Prior to the initiation of the study the three municipalities have signed a collaborative agreement, which in details describes each municipality’s role and contribution to the study, as well as duty of confidentiality, intellectual properties and publication rights. The trial will only be discontinued if the three municipalities, the Region of Southern Denmark or an ethical committee finds it necessary for regulatory or medical reasons.

#### Data collection

Data will be collected continuously on a weekly basis during the course of the study. In a typical week nurses in an intervention area home care team each receive a list of citizens at risk on a Monday morning. The nurses have two days (until Wednesday) to assess each citizen’s health situation using the ‘PATINA decision support tool’. On the fourth day (Thursday), the project manager (AF) carries out a weekly quality assessment of the gathered data. If data is missing or major errors are identified the project coordinator contacts the nurse responsible for the entered data to discuss the case, and if needed, adjust accordingly.

In accordance with Danish laws on data protection study data will be collected and stored in REDCap (version: REDCap 9.1.15 -© 2020 Vanderbilt University) [31, 32], an electronic data capture tool hosted at OPEN at Odense University Hospital, the Region of Southern Denmark. Data from the PATINA algorithm and decision support tool will be linked to data from Danish national health registries using the citizens unique social security number. The registers include data from the National Patient Registry (history of disease, diagnosis, acute or elective admissions, readmissions, outpatient care and hospital treatment), the National Health Insurance Service Register (services from primary care physicians, the Register of Medicinal Product Statistics, and the Danish Civil Registration System (age, gender, marital status and more).

### Data analyses

#### Sample size and statistical power

The three municipalities, Odense, Svendborg and Kerteminde, provide access to data on all citizens age 65+ and over, who receive home or nursing care, yielding an expected study population between 5.500–6.000 individuals.

We have calculated the power of the study using an assumption of a one-sided significance level of 0.05 and equal sample size between the intervention and control group. It has not been possible to identify previous studies investigating the effects of monitoring utilization of home care combined with an intervention to prevent acute admission. Thus, we use a conservative estimate of 10% difference in number of acute admissions between the intervention (10.8%) and control group (12%) [38]. On the basis of these assumptions the power in each of the three municipalities seems rather modest (Odense β = 0.15, Svendborg β = 0.12, Kerteminde β = 0.1). Across the three municipalities the power is somewhat greater, the β will at least be 0.22 depending on the timing.

The power calculation was carried out using STATA 16 and the command” .steppedwedge”, as described by Hemming and Taljaard [39].

#### Statistical analyses

Data management and statistical analyses will be carried out using SAS version 9.4 software (SAS Institute Inc., Cary, NC, USA) and STATA version 16 (StataCorp LLC, Texas, USA). The analyses will be supplemented by modelling and graphics using “R” software (Version 3.6.1) [25].

Data analyses will be conducted according to intention-to-treat principles and include all eligible patients with available outcome data. The analyses will be conducted according to the randomization schedule. Citizens’ characteristics will be reported using numbers/percentages, means (SD), and medians [IQR]. Differences between the intervention and control groups will be calculated using chi2 tests, Student’s t-test, or Kruskal-Wallis test, as appropriate. The statistical significance threshold for all tests will be set to *P* < 0.05.

When presenting the results of our study we will use suiting EQUATOR network guidelines such as the CONSORT 2010 statement [40] with the extension for stepped wedge cluster randomized trials [41].

#### Analysis of primary and secondary outcomes

Outcomes are shown in Table 2. The primary outcome, proportion of acute admission, will be analyzed using co-variance, repeated measure analysis and logistic regression. A key event is the switch from the usual care to intervention phase. We will treat the intervention regression coefficient as a slopes term to account for potential differences in intervention effects across area home care teams. The analyses will be adjusted for relevant confounders such as age, gender, co-morbidity, functional level and frailty.

For secondary outcomes (Table 2) 2, 3, 4, 5 and 6 we will use logistic regression to analyze occurrence of event. Citizens, who relocate to another municipality, move into a care home, or die during the study period will be excluded from the analyses. Models for acute admission will be used and the incidence of acute admission will be analyzed using logistic regression. The analysis will be adjusted for confounding factors such as age, gender and co-morbidity. We will stratify the analyses by municipalities and area home care teams to control for differences between these.

When analyzing readmission between the intervention and control group we will focus on both the 30-day readmission rate (outcome 3) and 90-day readmission rate (outcome 4). The index-admission will be defined as any admission to a Danish hospital during the study period. Citizens with multiple hospital admissions can have several index admissions but only one readmission per index admission.

Changes in citizens health service utilization (outcome 7, 8 and 9) will be evaluated in order to assess changes in costs of care and treatment in the primary and secondary health care sector. We will analyze the average cost per citizen, as well as analyses of the change in costs in primary and secondary health care. We will also analyze the costs of implementing the PATINA algorithm and decision support tool (outcome 10). This will be done by summing up the time spent on project related activities by both nurses and project coordinators in each municipality. The costs of implementing the algorithm will be reported as the total cost of the intervention with a 95% confidence interval.

Barriers and facilitators for nurses’ acceptance and use of the algorithm (outcome 11) will be analyzed using data gathered in a three-wave survey, which will be integrated in SW-RCT design and follow the step-wise implementation of the algorithm in each of the tree municipalities. When an area home care team is randomized to the intervention group the nurses allocated the section will complete a survey on three occasions; before the algorithm is implemented, just after having received training in the use of the algorithm, and after having worked with the algorithm for one month. The survey consists of several validated item scales within the areas of change management, motivation approach, intrinsic motivation, basic need satisfaction, public service motivation, technology acceptance and relational coordination. The analysis will aim to investigate the role of public professionals in achieving the desired effects of implementing algorithms in areas of public health administration and contributes to the broader public management and change management literature, by linking employees’ motivation and attitudes toward a change to the outcome of that change.

## Discussion

Demographic changes towards older populations pressure most industrialized countries health care systems to adapt integrated care models, as this may reduce the use of secondary health care services, but also means an expansion of the primary health care services [42]. To prevent the need for treatment in secondary health care, it is essential to provide sufficient treatment and care for older citizens, especially those who are frail, as this population have greater need for health care than younger citizens.

Even though it is still a novel area of research, prediction models developed to predict the risk of acute hospitalization among older community-dwelling citizens have been found to be effective in achieving predictive performance [18–20]. These models have primarily been developed based on health care data from electronic hospital records. To our knowledge the PATINA study will be one of the first studies to implement and test the effect of a prediction algorithm based on home care utilization in a real-life setting [3]. The algorithm and the tailored decision support tool will help nurses to systematically assess the health status and risk of acute hospitalization of older citizens. The expected benefits are reduced use of secondary health care services and improved patient outcomes. Further, we expect that the project will introduce a more detailed and suiting professional language for describing the unclear symptoms that may precede acute disease in older citizens. Such a language can be utilized both inside the municipal organization and in the cross-sectoral collaboration.

The stepped-wedge randomized controlled trial is well suited for studying and evaluating of service type interventions [43]. The random and sequential cross-over is pragmatic to implement as it mimics how large organizations involved in delivering health care service implement interventions. The stepped-wedge study design is especially relevant when evidence in support of an intervention already exist, as the intervention can be fully implemented after the completion of the study [43]. Further, the design is well suited for politically organized public authorities such as the Danish regions and municipalities, as it allows to incorporate rigorous scientific evaluations when implementing large interventions or changes without delaying the implementation process.

The sequential cross-over also allows researchers to study temporal effect with high accuracy compared to other cluster designs [43, 44]. However, in the PATINA study the cross-over must be handled with care as some area home care teams in Kerteminde and Svendborg municipalities are not geographically separated. Further, the design introduces a potential risk of secular trends unrelated to the PATINA algorithm and decision support tool. The study design also introduces a risk of unequal exposure to seasonal trends, as more citizens will be exposed to the algorithm in the end of the study [43, 44]. During the study we will keep track of changes and other interventions implemented in the three municipalities as well as on a regional and national level. Further, we will be aware and adjust for both the clustered design and confounding effect of time in our statistical analyses [41].

Like many other welfare states’ health care systems Danish regions and municipalities are economically incentivized to prevent unnecessary use of secondary health care services, especially acute admissions and readmission of older citizens. By completing an economic analysis focusing on the cost of changes in both municipal and regional health care utilization as well as cost of implementation it is our hope that we will be able to highlight potential economic incentives of introducing the PATINA algorithm and decision support tool. Further, by studying the barriers and facilitators for nurses’ acceptance and use of the ‘PATINA algorithm and decision support tool’, we hope to deliver key-insights at the managerial level when implementing interventions such as the PATINA algorithm.

### Study status

After achieving the necessary ethical and governance approvals the study began including area home care teams in the three Danish municipalities on the June 1, 2020 with an expected end date of May 31, 2021. The study start was postponed from April 1, 2020 (two months) due to COVID-19 and has run continuously since.

The study is registered at ClinicalTrials.gov (NCT04398797) and will be updated accordingly.

## Data Availability

Data sharing is not applicable at this point in the study. Whether data sharing will be possible after finishing the study in the future relies on obtaining permission from the Danish Data Protection Agency.

## Abbreviations

CONSORT: Consolidated Standards of Reporting Trials
COVID-19: COronaVIrus Disease 2019
OADR: Old Age Dependency Ration
PATINA: Prevention of AcuTe admIssioN Algorithm
REDCap: Research Electronic Data Capture
SPIRIT: Standard Protocol Items: Recommendations for Interventional Trial
SW-RCT: Stepped-Wedge Randomized Controlled Trial
